# Correction: Haddad, J.G., et al. *Doratoxylon apetalum*, an Indigenous Medicinal Plant from Mascarene Islands, Is a Potent Inhibitor of Zika and Dengue Virus Infection in Human Cells *Int. J. Mol. Sci.* 2019, *20*, 2382

**DOI:** 10.3390/ijms21217816

**Published:** 2020-10-22

**Authors:** Juliano G. Haddad, Andrea Cristine Koishi, Arnaud Gaudry, Claudia Nunes Duarte dos Santos, Wildriss Viranaicken, Philippe Desprès, Chaker El Kalamouni

**Affiliations:** 1 Université de la Réunion, INSERM U1187, CNRS UMR 9192, IRD UMR 249, Unité Mixte Processus Infectieux en Milieu Insulaire Tropical, Plateforme Technologique CYROI, 94791 Sainte Clotilde, La Réunion, France; juliano.haddad@univ-reunion.fr (J.G.H.); wildriss.viranaicken@univ-reunion.fr (W.V.); philippe.despres@univ-reunion.fr (P.D.); 2 Laboratorio de Virologia Molecular, Instituto Carlos Chagas, ICC/FIOCRUZ/PR, Curitiba, Parana 81350-010, Brazil; ackoishi@gmail.com (A.C.K.); clsantos@fiocruz.br (C.N.D.d.S.); 3 Bioactive Natural Products Unit, School of Pharmaceutical Sciences, EPGL, University of Geneva, Rue Michel-Servet 1, CH-1211 Geneva, Switzerland; Arnaud.gaudry@unige.ch

The author wishes to make the following correction to this paper [1]: In the Materials and Methods section, paragraph 3.2: Voucher specimen (RUN-055E) should be Voucher specimen (T-1097). The mistake was generated during final editing following peer-review. The mistake did not affect the review process. Our correction does not change the conclusions of this manuscript.

The authors would like to apologize for any inconvenience caused to the readers by these changes.
