# Supplementary material for: Doratoxylon apetalum, an Indigenous Medicinal Plant from Mascarene Islands, Is a Potent Inhibitor of Zika and Dengue Virus Infection in Human Cells
Source: Int J Mol Sci. 2019 May 14;20(10):2382. doi: 10.3390/ijms20102382 (PMC6567149; doi:10.3390/ijms20102382)
Supplement: Supplementary file 1 [file ijms-20-02382-s001.pdf]

Supplementary data

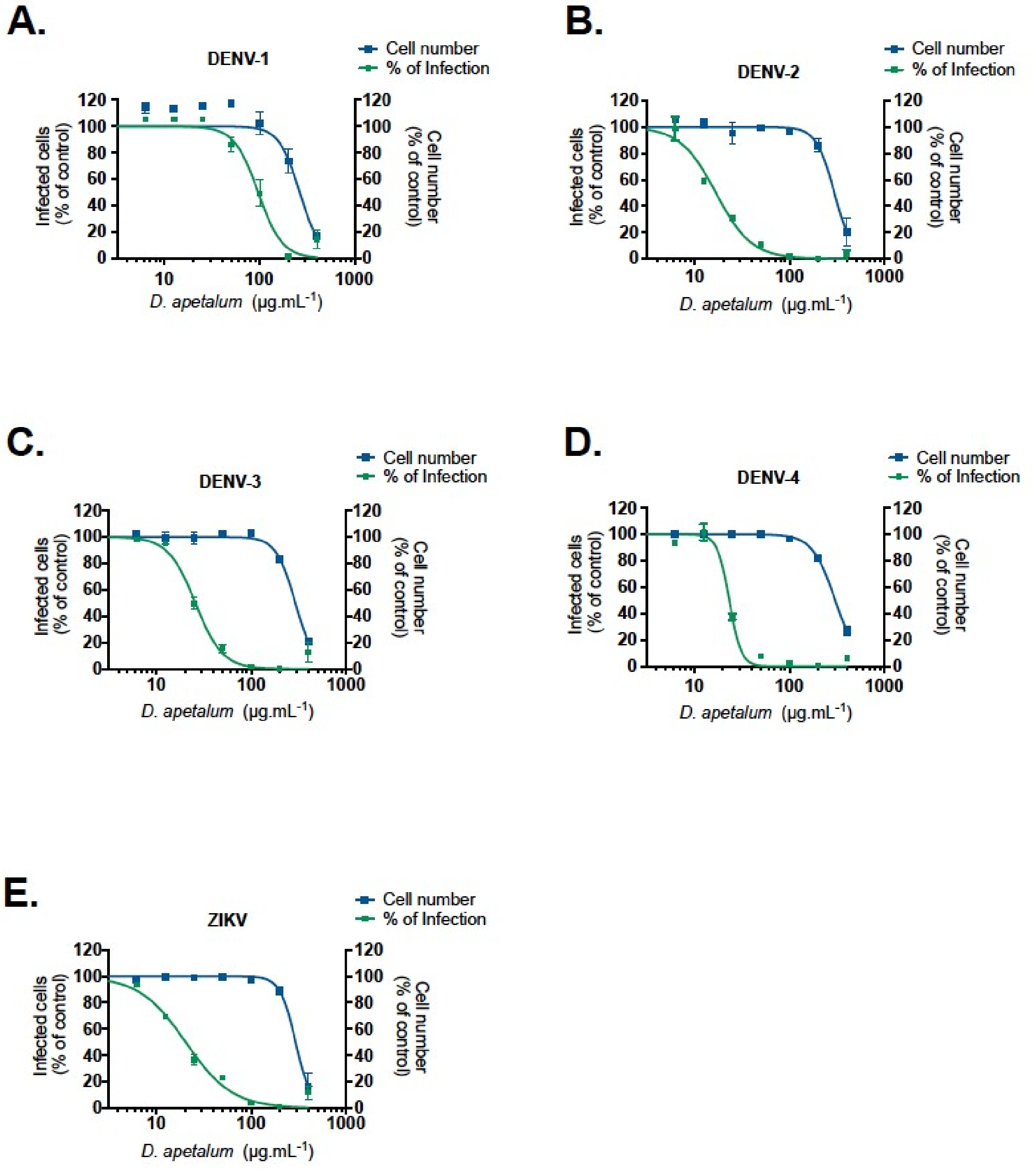

Figure S1: *D. apetalum* extract show a dose-dependent antiviral effect against four DENV clinical isolates and an epidemic Brazilian ZIKV strain. Huh7.5 cells were infected during 48 h with DENV at different MOI 2, 2, 0.5 and 2 for DENV 1-4 (**A, B, C, D**) respectively and MOI of 2 for Brazilian epidemic ZIKV strain (**E**). Cells were continuously incubated with different concentrations of *D. apetalum* extract for 48 h. The percentages of infected cells (E positive cells) and cell number were evaluated using the Operetta High-Content Imaging System. Results are means ± SD performed in triplicate and are expressed as relative values compared to Mock-infected cells.
